# Angiogenesis Dysregulation in Term Asphyxiated Newborns Treated with Hypothermia

**DOI:** 10.1371/journal.pone.0128028

**Published:** 2015-05-21

**Authors:** Henna Shaikh, Elodie Boudes, Zehra Khoja, Michael Shevell, Pia Wintermark

**Affiliations:** 1 Division of Newborn Medicine, Department of Pediatrics, McGill University, Montreal, Quebec, Canada; 2 Department of Neurology and Neurosurgery, McGill University, Montreal, Quebec, Canada; Ehime University Graduate School of Medicine, JAPAN

## Abstract

**Background:**

Neonatal encephalopathy following birth asphyxia is a major predictor of long-term neurological impairment. Therapeutic hypothermia is currently the standard of care to prevent brain injury in asphyxiated newborns but is not protective in all cases. More robust and versatile treatment options are needed. Angiogenesis is a demonstrated therapeutic target in adult stroke. However, no systematic study examines the expression of angiogenesis-related markers following birth asphyxia in human newborns.

**Objective:**

This study aimed to evaluate the expression of angiogenesis-related protein markers in asphyxiated newborns developing and not developing brain injury compared to healthy control newborns.

**Design/Methods:**

Twelve asphyxiated newborns treated with hypothermia were prospectively enrolled; six developed eventual brain injury and six did not. Four healthy control newborns were also included. We used Rules-Based Medicine multi-analyte profiling and protein array technologies to study the plasma concentration of 49 angiogenesis-related proteins. Mean protein concentrations were compared between each group of newborns.

**Results:**

Compared to healthy newborns, asphyxiated newborns not developing brain injury showed up-regulation of pro-angiogenic proteins, including fatty acid binding protein-4, glucose-6-phosphate isomerase, neuropilin-1, and receptor tyrosine-protein kinase erbB-3; this up-regulation was not evident in asphyxiated newborns eventually developing brain injury. Also, asphyxiated newborns developing brain injury showed a decreased expression of anti-angiogenic proteins, including insulin-growth factor binding proteins -1, -4, and -6, compared to healthy newborns.

**Conclusions:**

These findings suggest that angiogenesis pathways are dysregulated following birth asphyxia and are putatively involved in brain injury pathology and recovery.

## Introduction

In angiogenesis, a coordinated orchestra of proteins causes new blood vessels to sprout and mature from existing vessels. This process begins with the degradation of the local extracellular matrix and the weakening of tight junctions and interactions between endothelial cells and pericytes. Vascular destabilization permits endothelial cell proliferation and migration to a site of nascent tube formation. Angiogenesis is completed as intercellular interactions and the extracellular matrix are re-established to stabilize the new blood vessels [1]. Recent studies of adult stroke highlight angiogenesis as a potential therapeutic target. Enhanced angiogenesis is accompanied by enhanced neurogenesis and improved neurological recovery in animal models of adult stroke [2–4] and is correlated with improved outcomes in human adults [5].

Neonatal encephalopathy is a major predictor of neonatal death and long-term neurological deficits, including cerebral palsy, intellectual disability, and epilepsy [6]. Currently, neonatal encephalopathy attributed to asphyxia is treated with mild hypothermia, which has been shown to reduce mortality and morbidity in clinical trials [7]. However, hypothermia must be initiated within 6 hours of life, requires cumbersome equipment, and shows decreased benefits for severely asphyxiated newborns [8]; thus more robust and versatile treatment options are needed. While hypothermia protects the brain from acute damage due to hypoxia-ischemia, enhancing angiogenesis may help to restore the neurovascular niche, facilitating optimal neuronal and glial re-growth in the sub-acute and chronic phases of hypoxia-ischemia [9, 10]. A few angiogenic markers have been studied in asphyxiated newborns and in rodent models of neonatal encephalopathy; these studies have demonstrated increased expression of VEGF, its receptors, and other angiogenic proteins following the injury [11–15]. However, to date, no systematic study examines the broader expression of angiogenesis-related markers following asphyxia injury in human newborns.

We hypothesized that angiogenesis is activated following birth asphyxia, and that this activation may differ between asphyxiated newborns developing and not developing brain injury. Thus, the objective of this study was to evaluate the expression of angiogenesis-related protein markers in asphyxiated newborns treated with hypothermia developing and not developing brain injury compared to healthy control newborns.

## Methods

### Patients

We conducted a cohort study of term asphyxiated newborns admitted to our neonatal intensive care unit who met the criteria for induced hypothermia [16–18]: (1) gestational age ≥ 36 weeks and birth weight ≥ 2000 g; (2) evidence of fetal distress, e.g. history of an acute perinatal event, cord pH ≤ 7.0 or base deficit ≥ 16 mEq/L; (3) evidence of neonatal distress, such as an Apgar score ≤ 5 at 10 minutes, postnatal blood gas pH obtained within the first hour of life ≤ 7.0 or base deficit ≥ 16 mEq/L, or a need for ventilation initiated at birth and continued for at least 10 minutes; and (4) evidence of moderate to severe neonatal encephalopathy as evident by an abnormal standard neurological exam and/or amplitude-integrated electroencephalogram. Eligible patients received whole-body cooling to an esophageal temperature of 33.5°C initiated within the first 6 hours of life, continued for 72 hours, and followed by slow rewarming.

Clinical data, including gestational age, birth weight, sex, Apgar score at 10 minutes, use of intubation and chest compression at birth, arterial cord pH, and initial infant blood gas pH were collected prospectively for each asphyxiated newborn. The hour of life at which hypothermia was initiated was also calculated and recorded.

Healthy term newborns with normal brain MRI findings were included as healthy controls. The research protocol was approved by the research ethics board from the Montreal Children’s Hospital, McGill University Health Centre, and informed written consent was obtained from the parents on behalf of their newborns in all cases.

### Imaging

The presence or absence of brain injury in asphyxiated and healthy control newborns was defined by magnetic resonance imaging obtained around day 10 of life (range: day 9–13 of life), as they have previously been reported to define precisely the extent of the brain injury in these newborns [19–21]. Neuroradiologists blinded to the newborns’ clinical condition reviewed the images and scored them using a previously described system for evaluating brain injury in asphyxiated newborns [22]. For the purpose of this study, asphyxiated newborns treated with hypothermia were then classified as “with” or “without injury” depending on whether or not any brain injury was observed on the magnetic resonance imaging.

### Angiogenesis-related proteins

Nurses collected blood samples in tubes containing potassium EDTA at 24 hours of life for healthy control newborns and at 6, 24, 48, 72, and 96 hours of life for asphyxiated newborns treated with hypothermia. Blood samples were immediately centrifuged at 3600 rpm for 6 minutes at room temperature. Following centrifugation, plasma was removed, aliquotted, and stored at -80°C until tested. Plasma samples were coded, and protein markers were measured blindly with respect to patient identity and disease.

The samples were thawed at room temperature, vortexed, spun at 13,000 x g for 5 minutes for clarification and 40 mcL were transferred to a master microtiter plate for multi-analyte profiling (MAP) antigen analysis. Using automated pipetting, an aliquot of each sample was introduced into one of the capture microsphere multiplexes of the Rules-Based Medicine (Myriad RBM) Custom Human multi-analyte profile (MAP) (Myriad, Austin, TX) (http://www.rules-basedmedicine.com). These mixtures of sample and capture microspheres were thoroughly mixed and incubated at room temperature for 1 hour. Multiplexed cocktails of biotinylated, reporter antibodies for each multiplex were then added robotically and, after thorough mixing, were incubated for an additional hour at room temperature. Multiplexes were developed using an excess of streptavidin-phycoerythrin solution, which was thoroughly mixed into each multiplex and incubated for 1 hour at room temperature. The volume of each multiplexed reaction was reduced by vacuum filtration and the volume increased by dilution into matrix buffer for analysis. Analysis was performed in a Luminex 100 instrument and the resulting data stream was interpreted using proprietary data analysis software developed at Rules-Based Medicine (Myriad RBM). For each multiplex, both calibrators and controls were included on each microtiter plate. 8-point calibrators were run in the first and last column of each plate and 3-level controls were included in duplicate. Testing results were determined first for the high, medium and low controls for each multiplex to ensure proper assay performance. Unknown values for each of the analytes localized in a specific multiplex were determined using 4- and 5- parameter weighted and non-weighted curve fitting algorithms included in the data analysis package. Expression of 49 angiogenesis-related proteins was thus analyzed. Analyzed proteins, listed in “Table 1”, were selected based on angiogenic involvement and assay availability; the different pathways in which they act are schematically represented in “S1 Fig”. Plasma concentrations were reported for each sample unless the sample quantity was not sufficient (QNS), the concentration was below the lower limit of quantification (LLOQ), or the concentration was above the highest quantifiable value.

**Table 1:** Angiogenesis-related protein markers analyzed in the study.

Vascular Destabilization	Endothelial cells survival, proliferation and migration		Neurogenesis and angiogenesis	Anti-angiogenesis	Apoptosis
Angiopoietin-2 (Ang-2)	Angiogenin (Ang)	Kallikrein 5 (KLK-5)	Brain-Derived Neurotrophic Factor (BDNF)	Endostatin	Fas Ligand (FasL)
Cathepsin D	AXL Receptor Tyrosine Kinase (AXL)	Macrophage-Stimulating Protein (MSP)	Neuron-Specific Enolase (NSE)	Fibulin-1C (Fib-1C)	FasL Receptor (FasR)
Hepsin	Endoglin	Neuropilin-1 (NP-1)	Neuronal Cell Adhesion Molecule (Nr-CAM)	Human Epididymis Protein 4 (HE4)	Sortilin
Matrix Metalloproteinase-3 (MMP-3)	Epidermal Growth Factor Receptor (EGFR)	Receptor tyrosine-protein kinase erbB-3 (ErbB3)		Insulin-like Growth Factor-Binding Protein 1 (IGFBP-1)	TNF-Related Apoptosis-Inducing Ligand Receptor 3 (TRAIL-R3)
Matrix Metalloproteinase-7 (MMP-7)	Fatty Acid-Binding Protein 4, Adipocyte (FABP-4)	Stem Cell Factor (SCF)		Insulin-like Growth Factor Binding Protein 4 (IGFBP4)	
Matrix Metalloproteinase-9 (MMP-9)	Galectin-3	Superoxide Dismutase 1, soluble (SOD-1)	**Vascular stabilization**	Insulin-like Growth Factor Binding Protein 5 (IGFBP5)	
Matrix Metalloproteinase-10 (MMP-10)	Glucose-6-phosphate Isomerase (G6PI)	Tenascin-C (TN-C)	Cellular Fibronectin (cFib)	Insulin-like Growth Factor Binding Protein 6 (IGFBP6)	
	Heparin-Binding EGF-Like Growth Factor (HB-EGF)	Tumor necrosis factor receptor 2 (TNFR2)	Collagen IV	Tissue Inhibitor of Metalloproteinases 1 (TIMP-1)	
	Human Epidermal Growth Factor Receptor 2 (HER-2)	Vascular Endothelial Growth Factor C (VEGF-C)	Tyrosine kinase with Ig and EGF homology domains 2 (TIE-2)		
	Insulin-like Growth Factor-Binding Protein 2 (IGFBP-2)	Vascular Endothelial Growth Factor Receptor 2 (VEGFR-2)			
	Insulin-like Growth Factor-Binding Protein 3 (IGFBP-3)	Vascular endothelial growth factor receptor 3 (VEGFR-3)			
	Intercellular Adhesion Molecule 1 (ICAM-1)	YKL-40			

The 49 proteins analyzed in this study are categorized according to their predominant role in angiogenesis. Obviously, many proteins serve multiple, context-dependent functions and may fit into more than one category.

### Data analysis

Protein expression data were represented as mean concentration ± standard deviation for each group of newborns. To test differences in protein expression between groups, we used Mann-Whitney U tests. A *p* value *< 0*.*05* was used to highlight protein expression differences between groups. All analyses were performed with SPSS Version 20.0 for Windows (SPSS Inc., Chicago, IL, USA). For graphical representation, ratios of group means were calculated and log transformed to obtain fold change data for each protein marker. In addition, to improve data interpretation, a DAVID functional clustering of Gene Ontology Terms was performed [23–24].

## Results

### Patients

**Fig 1:**
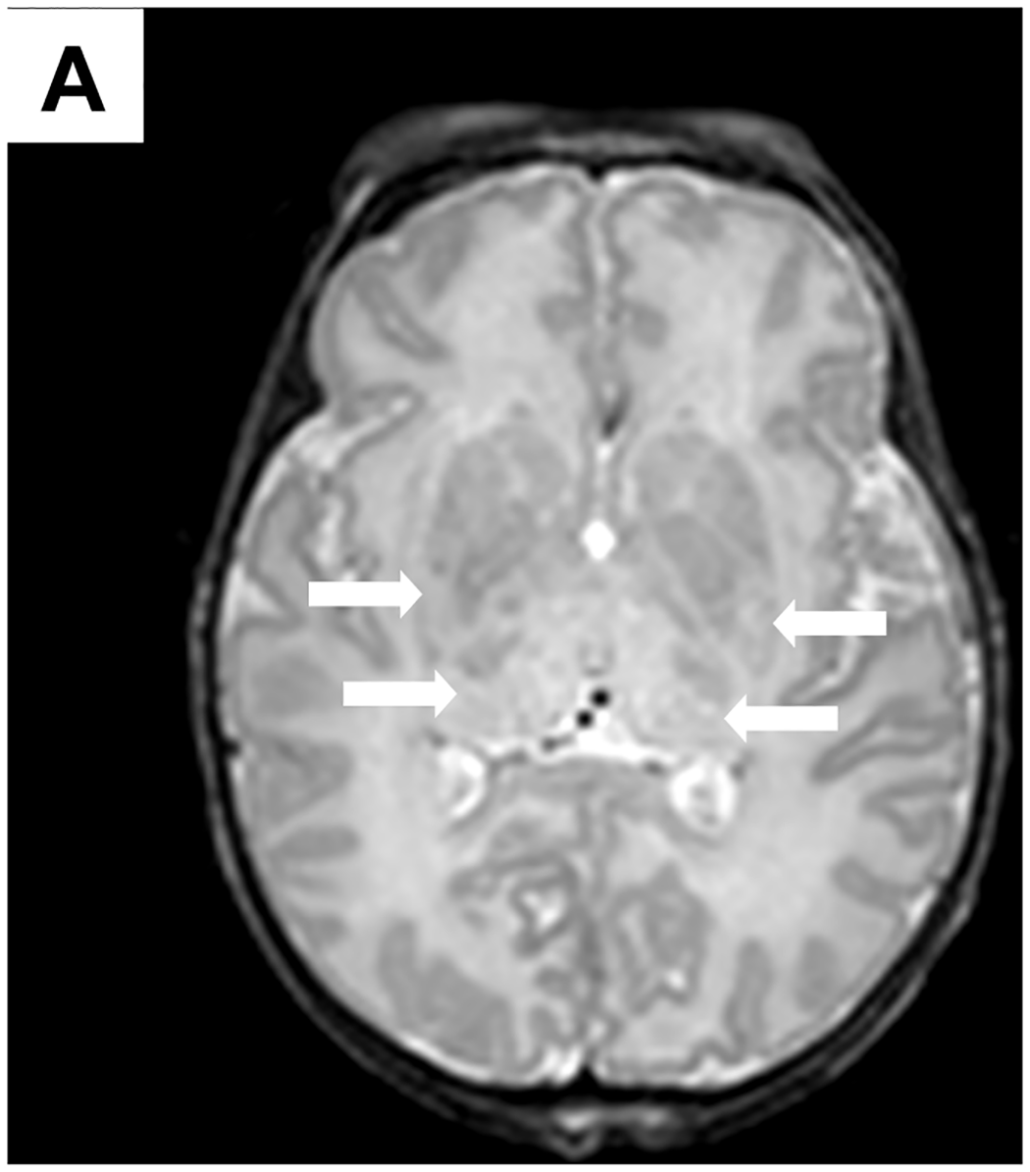
Brain MRIs of a term asphyxiated newborn treated with hypothermia, performed on day 9 of life. **(A)** The T2-weighted imaging shows the injury within the thalami and lentiform nuclei (arrows).

Twelve asphyxiated newborns treated with hypothermia and four healthy control newborns were included in this study. Six of the asphyxiated newborns developed brain injury; four developed a basal ganglia injury pattern (“Fig 1”) and two a watershed injury pattern. The remaining six newborns did not develop any brain injury. Findings on brain MRI of the healthy control newborns were normal. Clinical characteristics of all asphyxiated newborns treated with hypothermia are shown in “Table 2”. Gestational age, birth weight, sex, Apgar score at 10 minutes, use of intubation and chest compression at birth, arterial cord pH, and initial blood gas pH were not different between the asphyxiated newborns developing and not developing brain injury.

**Table 2:** Clinical characteristics of the asphyxiated newborns treated with hypothermia.

Variables	Healthy newborns (n = 4)	All asphyxiated newborns (n = 12)	Asphyxiated newborns treated with hypothermia not developing brain injury (n = 6)	Asphyxiated newborns treated with hypothermia developing brain injury (n = 6)	*p value*
**Clinical characteristics**					
Gestational age (weeks), mean ± SD	39.39 ± 0.85	39.08 ± 1.89	38.86 ± 184	39.31 ± 2.09	*0*.*63*
Birth weight (g), mean ± SD	3412 ± 525	3131 ± 608	3183 ± 276	3080 ± 855	*0*.*87*
Sex					*0*.*51*
Male, n(%)	1 (25)	9 (75)	5 (83)	4 (67)	
Female, n(%)	3 (75)	3 (25)	1 (17)	2 (33)	
Apgar score ≤ 5 at 10 minutes, n(%)	0 (0)	9 (75)	5 (83)	4 (67)	*0*.*51*
Intubation at birth, n(%)	0 (0)	11 (92)	6 (100)	5 (83)	*0*.*30*
Chest compression at birth, n(%)	0 (0)	7 (58)	4 (67)	3 (50)	*0*.*56*
Arterial cord pH, mean ± SD	-	6.96 ± 0.18	7.03 ± 0.13	6.87 ± 0.21	*0*.*33*
Initial postnatal blood gas pH, mean ± SD	-	7.06 ± 0.19	7.11 ± 0.15	7.03 ± 0.21	*0*.*61*
Initiation of hypothermia (hours), mean ± SD	-	5.17 ± 0.86	5.29 ± 0.88	5.06 ± 0.92	*0*.*69*

### Expression of angiogenesis-related proteins at 24 hours of life

**Fig 2:**
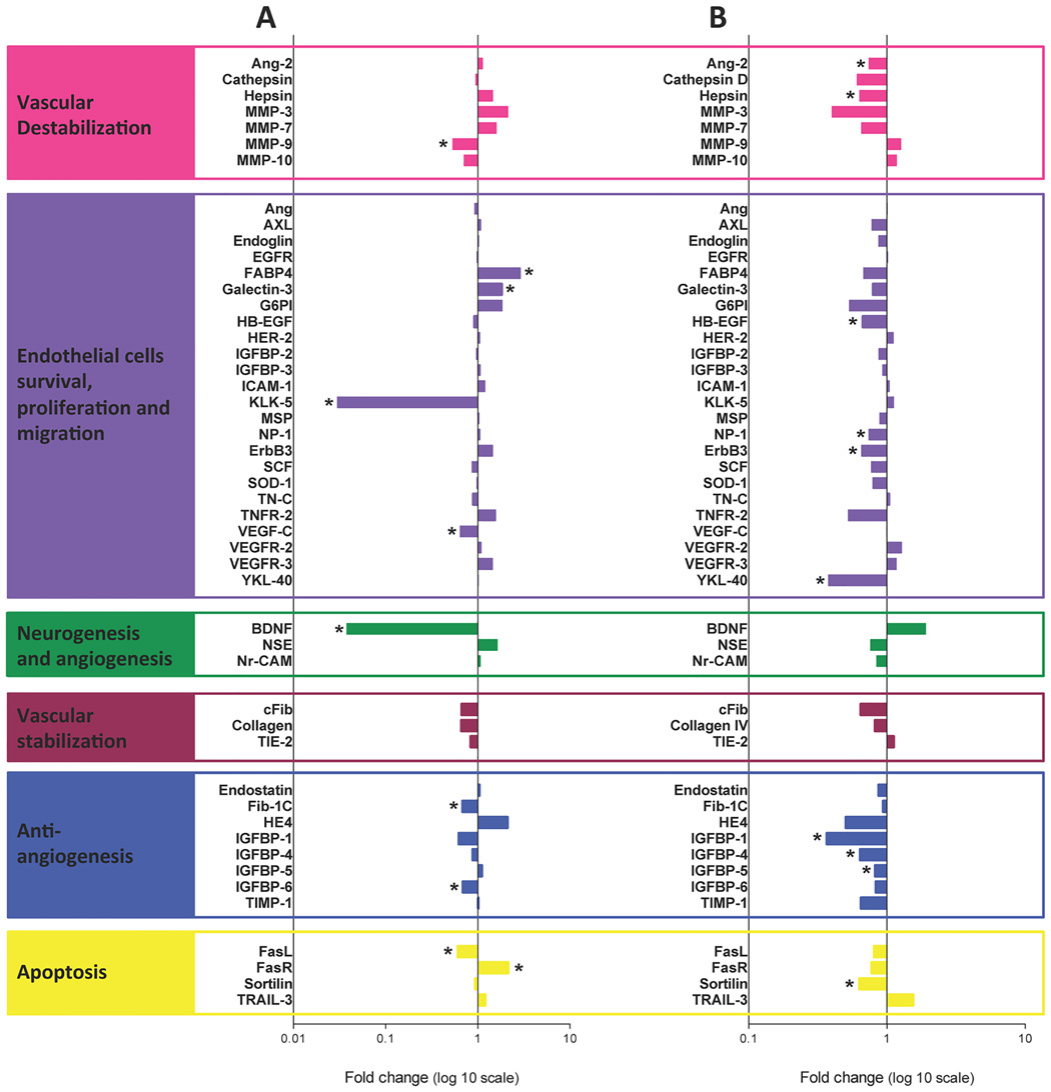
Expression of angiogenesis-related protein markers at 24 hours of life. Mean concentrations in each group were compared. *p* values were calculated to highlight significant protein expression differences between the groups (* *p < 0*.*05)*. For graphical representation, ratios between group mean concentrations were calculated and log transformed to obtain fold change data for each marker. **(A)** Fold changes in protein expression between asphyxiated newborns treated with hypothermia and healthy newborns. **(B)** Fold changes in protein expression between asphyxiated newborns developing and not developing brain injury.

Mean protein concentration values were compared between asphyxiated newborns developing and not developing brain injury and healthy newborns at 24 hours of life (“Fig 2”).

Asphyxiated newborns treated with hypothermia showed altered expression in 10 of the 49 angiogenesis-related proteins compared to healthy newborns (“Fig 2A”). Matrix metalloproteinase-9 (MMP-9), which is important for degrading the extracellular matrix, was decreased. Fatty acid binding protein-4 (FABP-4) and galectin-3 (Gal-3), which enhance endothelial cell survival, proliferation and migration, were up-regulated among asphyxiated newborns, while kallikrein-5 (KLK-5) was down-regulated. VEGF-C, which promote endothelial cell permeability along with survival and proliferation, was decreased. Brain derived neurotrophic factor (BDNF), which has a dual impact on neurogenesis and angiogenesis, was down-regulated. The anti-angiogenic proteins fibulin-1C (Fib-1C) and insulin-like growth factor-binding protein-6 (IGF-BP-6) were down-regulated in asphyxiated newborns. Finally, while the apoptotic protein Fas ligand (FasL) showed decreased expression, its receptor (FasR) showed increased expression. When performing a DAVID functional clustering of Gene Ontology Terms using the 49 angiogenesis-related proteins as background and the abovementioned 10 dysregulated proteins as the Gene List, one cluster of proteins stood out all related to extracellular signaling activities (MMP-9, KLK-5, Fib-1C and IGF-BP6); six proteins could not be grouped.

When comparing asphyxiated newborns developing and not developing brain injury at 24 hours of life, the expression of 10 angiogenesis-related proteins was decreased in the prior group compared to the latter (“Fig 2B”**)**. Two of these proteins, angiopoietin-2 (Ang-2) and hepsin destabilize the vasculature to permit new sprouting. Expression of heparin-binding epidermal growth factor-like growth factor (HB-EGF), which is important for endothelial cell survival, proliferation, and migration, was decreased. Neuropilin-1 (NP-1), receptor tyrosine-protein kinase erbB-3 (Erb-B3), and YKL-40, which promote similar endothelial cell effects, also showed diminished expression. Expression of the angiogenic inhibitors IGF-BP-1, IGF-BP-4, and IGF-BP-5 and the apoptotic protein sortilin was also decreased. When performing a DAVID functional clustering of Gene Ontology Terms using the 49 angiogenesis-related proteins as background and the abovementioned 10 dysregulated proteins as the Gene List, one cluster of proteins stood out all related to extracellular signaling activities (Ang-2, Erb-B3, HB-EGF, IGF-BP-1, IGF-BP-4, IGF-BP-5, NP-1, YKL-40); one protein could not be grouped.

Comparisons in protein expression between asphyxiated newborns not developing brain injury and healthy newborns, as well as between asphyxiated newborns developing brain injury and healthy newborns were included in “S2 Fig”.

### Evolution of angiogenesis-related protein expression in the first days of life

**Fig 3:**
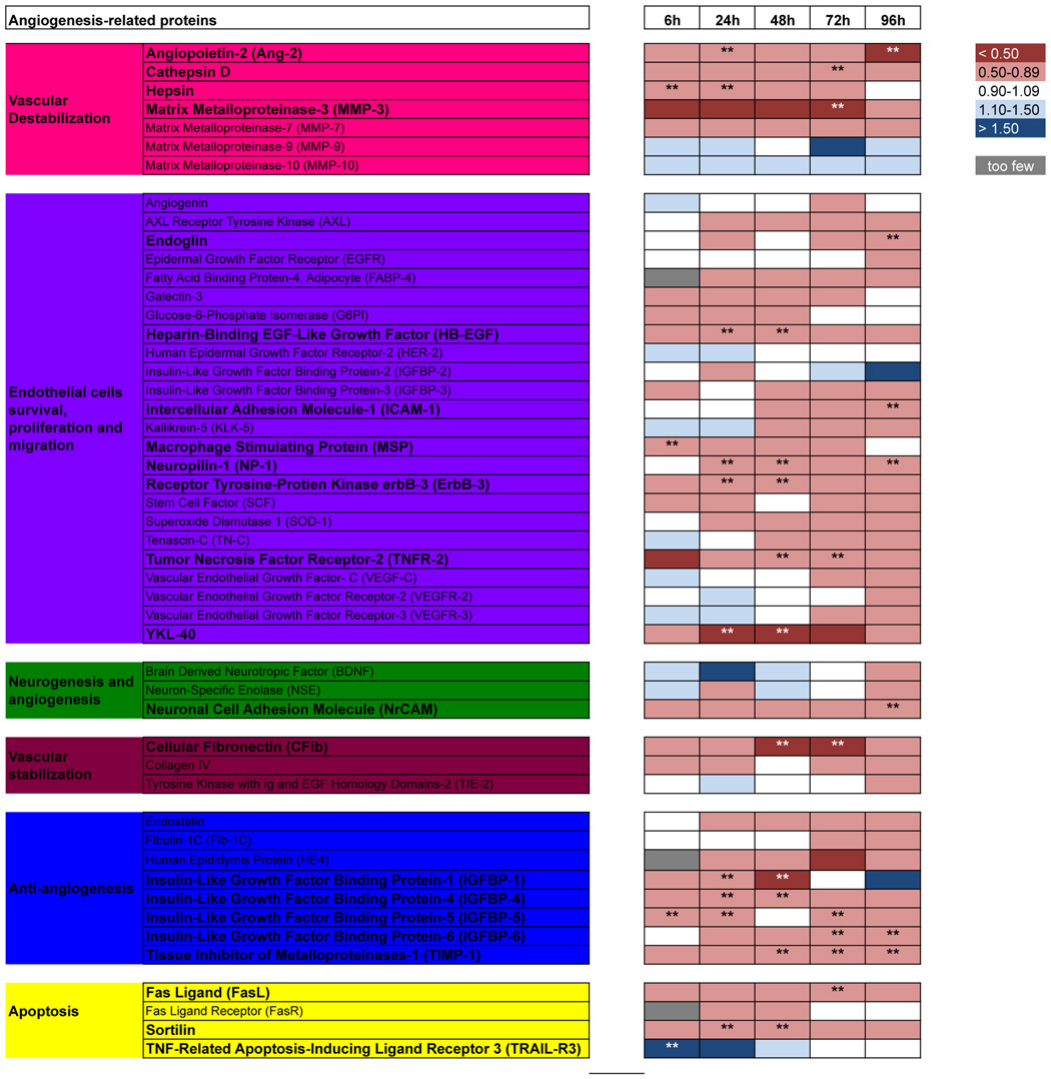
Evolution of angiogenesis-related protein expression over time in asphyxiated newborns developing brain HI injury compared to asphyxiated newborns not developing brain HI injury. The heat map depicts the fold changes in protein concentration between the two groups. Fold change ranges are denoted by the color coding in the legend at the right. ** *p < 0*.*05*.

Mean protein concentration values were also compared between asphyxiated newborns developing and not developing brain injury throughout the first days of life, i.e., at 6, 24, 48, 72, and 96 hours of life (“Fig 3”). Among the vessel destabilizing proteins, hepsin expression was decreased early after asphyxia at 6 and 24 hours of life in newborns developing brain injury. Cathepsin-D (Cat-D) and MMP-3 expression decreased later at 72 hours. Angiopoietin-2 showed an early and late decrease at 24 and 96 hours. Among other proteins like hepsin, involved in endothelial cell survival, proliferation, and migration, macrophage-stimulating protein (MSP) showed an early decrease at 6 hours of life. HB-EGF, NP-1, Erb-B3, and YKL-40 were decreased at 24 and 48 hours of life, and NP-1 was also decreased at 96 hours of life. Tumor necrosis factor receptor-2 (TNFR-2) was decreased at 48 and 72 hours of life. Later, at 96 hours of life, endoglin, intercellular adhesion molecule-1 (ICAM-1) and neuronal cell adhesion molecule (NrCAM) were decreased. Among vessel stabilizing proteins, cellular fibronectin (cFib) expression was decreased at 48 and 72 hours of life. The anti-angiogenic protein IGF-BP-5 showed an early decrease at 6 hours of life, which was maintained at 24 and 72 hours of life. IGF-BP-1 and IGF-BP-4 expression decreased at 24 and 48 hours of life. IGF-BP-6 showed a later decrease in expression at 72 and 96 hours of life. Another anti-angiogenic protein, tissue inhibitor of metalloproteinases-1 (TIMP-1), was also decreased at 48, 72, and 96 hours of life. Finally, among apoptotic proteins, tumor necrosis factor-related apoptosis-inducing ligand receptor-3 (TRAIL-R3) showed an early increase at 6 hours of life, sortilin was decreased at 24 and 48 hours of life and FasL expression was decreased at 72 hours of life.

## Discussion

This is the first systematic study of the expression of angiogenesis-related protein markers following birth asphyxia in human newborns. The expression of angiogenesis-related proteins was clearly dysregulated in asphyxiated newborns treated with hypothermia compared to healthy newborns, suggesting that angiogenesis plays an important but under-recognized role following birth asphyxia.

Interestingly, asphyxiated newborns who did not develop brain injury showed increased expression of four proteins involved in endothelial cell survival, proliferation, and migration (i.e., FABP4, G6PI, NP-1 and Erb-B3) compared to healthy newborns, while only one such protein (i.e., Gal3) was up-regulated in asphyxiated newborns developing brain injury. In comparison to asphyxiated newborns not developing brain injury, those who did develop an injury showed decreased expression of HB-EGF, NP-1, Erb-B3 and YKL-40, four proteins that promote endothelial cell survival, proliferation, and migration. This suggests that up-regulation of pro-angiogenic proteins may be neuroprotective or neurorestorative in asphyxiated newborns. Previous studies examining the individual roles of these proteins support their isolated effects on neuroprotection and neurorestoration. For example, animal models of adult stroke show that NP-1 facilitates VEGF signaling to promote neurorestoration [25]. HB-EGF-related neuroprotection has also been demonstrated in adult rodents subjected to experimental stroke [26–28]. Additionally, increased Gal3 expression has been found in rodents following experimental hypoxia-ischemia [29] and in the cerebrospinal fluid of asphyxiated newborns, where greater expression was correlated with a worse outcome [30].

Intriguingly, asphyxiated newborns developing brain injury showed decreased expression of four anti-angiogenic proteins (i.e., Fib-1C, IGF-BP-1, IGF-BP-4, and IGF-BP-6) compared to healthy newborns, while only two of these proteins (i.e., Fib-1C and IGF-BP-6) were down-regulated in asphyxiated newborns not developing brain injury compared to healthy newborns. In addition, IGF-BP-1, -4, and -5 were less expressed in asphyxiated newborns developing brain injury compared to those not developing brain injury. Evidence suggests that IGF-BP-1, -4, -5 and -6, which bind IGF-1, inhibit angiogenesis [31–33]. While no previous studies have examined the expression of the IGF-BPs after neonatal encephalopathy, animal models of neonatal hypoxia-ischemia demonstrate conflicting findings on IGF-1 expression after the insult [34–37], and decreased serum IGF-1 has been reported in asphyxiated newborns [38]. The decreased expression of IGF-BPs and other anti-angiogenic proteins observed in the present study may represent a mechanism for compensating for a failure to adequately up-regulate pro-angiogenic proteins in asphyxiated newborns developing brain injury.

In this study, protein expression was also altered in other steps of the angiogenesis pathway, beginning with vascular destabilization. MMP-9 expression was decreased among asphyxiated newborns not developing brain injury. Previous studies have found that MMP-9, which degrades the extracellular matrix to facilitate vascular sprouting [39], is increased following hypoxia-ischemia in rodent models [40–42] and human newborns [42–43]. However, in another study, no change was found in cerebrospinal fluid MMP-9 concentration following birth asphyxia in human newborns [30]. Beyond facilitating angiogenesis, this increase in MMP-9 may contribute to brain injury via blood brain barrier (BBB) disruption and neuroinflammation [40]. Therefore, the decrease in MMP-9 expression presently observed among asphyxiated newborns not developing brain injury may reflect an endogenous neuroprotective or neurorestorative mechanism or an effect of the hypothermia treatment. Ang-2 and hepsin are also involved in extracellular matrix degradation and promote neovascular sprouting in angiogenesis [44–45]. Both Ang-2 and hepsin were expressed at lower levels in asphyxiated newborns developing brain injury, suggesting that decreased expression of these proteins may contribute to injury pathology.

VEGF-C expression was decreased in all asphyxiated newborns, which may represent a mechanism for protecting against vascular permeability-related edema [46–48]. This is in contrast with data in rodent models demonstrating increased VEGF expression following hypoxia-ischemia [12, 13, 40, 49] and a protective effect of VEGF administration following the insult [50–51]. However, VEGF findings in human newborns vary more widely. While a few studies have found increased VEGF expression in cord blood [52] and cerebrospinal fluid [53], others have found either no difference in cerebrospinal fluid VEGF levels between asphyxiated and healthy newborns [54] or lower serum VEGF expression in severely asphyxiated compared to healthy newborns [55]. In the present study, KLK-5 expression was reduced in all asphyxiated newborns; KLK-5 promotes extracellular matrix degradation and neovascular sprouting by increasing MMP-9 activity, which, as previously noted, may contribute to brain injury [40, 56]. Decreased KLK-5 expression may therefore represent another possible mechanism of neuroprotection or neurorestoration.

This study also demonstrated decreased BDNF expression in all asphyxiated newborns. Previously, in studies of asphyxiated human newborns, increased BDNF levels have been found in cord blood at birth [57], blood serum 24 hours after birth [58], and cerebrospinal fluid [59], with the degree of increased expression correlated to brain injury severity [58]. Increased brain BDNF expression was also found in a rat model of neonatal hypoxia-ischemia [60]. Decreased BDNF expression may thus again reflect a neuroprotective or neurorestorative response or an effect of the hypothermia treatment.

Among the proteins important for vascular stabilization, collagen IV expression was decreased in asphyxiated newborns developing brain injury compared to healthy newborns. No previous studies of collagen IV expression following birth asphyxia exist; however, this protein is an essential component of the extracellular matrix, and its breakdown is necessary for new vessel sprouting. Therefore, decreased collagen IV expression may again indicate some compensatory mechanism for a failure to adequately up-regulate pro-angiogenic proteins in asphyxiated newborns developing brain injury.

Among apoptotic proteins, FasR was up-regulated in all asphyxiated newborns while FasL was down-regulated in asphyxiated newborns developing brain injury compared to healthy newborns. Additionally, sortilin expression was decreased in asphyxiated newborns developing brain injury compared to those not developing brain injury. No previous studies of serum concentrations of these proteins in asphyxiated newborns exist; however, Fas signaling has been shown to contribute to brain injury following neonatal hypoxia-ischemia [61].

This study was intended to highlight whether angiogenesis pathways are preferentially activated following birth asphyxia. As per the DAVID functional clustering of Gene Ontology Terms, the dysregulated angiogenesis-related proteins were mostly involved in extracellular signaling activities, but also in neurogenesis and programmed cell death. Given that this study demonstrates a significant role for angiogenesis after birth asphyxia, further investigation with larger sample sizes and targeted analysis of angiogenesis-related protein expression is definitively warranted. An important next step also includes validation of the dysregulated proteins using a distinct technique (e.g. Western blot). Such investigation may lead to the discovery of potential novel therapeutic targets. It would have also been ideal to study a group of asphyxiated newborns not treated with hypothermia so to distinguish how the hypothermia treatment rather than the asphyxia influenced some of the observed changes. However, as cooling is now the standard of care, it is no longer ethically possible to not receive hypothermia to assess the evolution over time of these angiogenesis-related proteins.

In this study, systemic plasma samples were analyzed rather than brain-specific samples. While studying cerebrospinal fluid would have provided a more direct view of the brain’s angiogenic response, ethical and practical concerns make it difficult to collect serial cerebrospinal fluid samples in sick newborns. Birth asphyxia has global effects on the newborn body, so altered plasma protein expression reflects peripheral as well as brain injuries [42]. Nonetheless, birth asphyxia does appear to disrupt the blood-brain barrier [62], which may release proteins from the brain into the peripheral systemic circulation. A final limitation of any study of neonatal encephalopathy in human newborns is the difficulty of accurately determining the time and duration of the asphyxial insult, which somewhat compromises the reliability of comparisons between newborns. Despite this, the current study demonstrates that angiogenic pathways appear to be activated at a similar time in the different newborns.

## Conclusions

In conclusion, this study highlights that angiogenesis pathways are dysregulated following birth asphyxia and are putatively involved in brain injury pathology and recovery. Our results suggest that a failure to up-regulate pro-angiogenic proteins may leave these newborns more susceptible to eventual brain injury. Furthermore, those newborns who fail to up-regulate pro-angiogenic proteins may attempt to compensate by down-regulating anti-angiogenic proteins. Overall, it seems that angiogenesis enhancement may be an important determinant of injury outcome following birth asphyxia. These findings may have future therapeutic applications to optimize eventual recovery following asphyxia and should be further investigated.

## Supporting Information

S1 Fig Schematic representation of the different studied angiogenesis-related pathways. Expression of 49 angiogenesis-related proteins, which were selected based on angiogenic involvement and assay availability, was analyzed.
(TIF)

S2 Fig Expression of angiogenesis-related protein markers at 24 hours of life. Mean concentrations in each group were compared. *p* values were calculated to highlight significant protein expression differences between the groups (* *p < 0*.*05)*. For graphical representation, ratios between group mean concentrations were calculated and log transformed to obtain fold change data for each marker. **(A) Fold changes in protein expression between asphyxiated newborns not developing brain injury and healthy newborns**. Eleven angiogenesis-related proteins showed altered expression. MMP-9, KLK-5, VEGF-C and BDNF levels remained reduced; however no change was evident in the levels of Gal-3. FABP-4 expression remained elevated. In addition, a greater number of proteins enhancing endothelial cell survival, proliferation and migration were up-regulated among asphyxiated newborns not developing brain injury. Expression of neuropilin-1 (NP-1), which is a VEGF receptor-2 (VEGFR-2) co-receptor, was increased. The expression of other proteins important for endothelial cell survival, proliferation, and migration, including glucose-6-phosphate isomerase (G6PI) and receptor tyrosine-protein kinase erbB-3 (Erb-B3), was also increased. The anti-angiogenic proteins Fib-1C and IGF-BP-6 remained decreased. Among the apoptotic proteins, no change was apparent in FasL expression; however FasR expression remained increased. When performing a DAVID functional clustering of Gene Ontology Terms using the 49 angiogenesis-related proteins as background and the abovementioned 11 dysregulated proteins as the Gene List, two clusters of proteins stood out, one related to neurogenesis (BDNF, NP-1, Erb-B3, VEGFC) and one related to extracellular signaling activities (Fib-1C, IGF-BP-6, KLK-5, MMP-9); three proteins could not be grouped. **(B) Fold changes in protein expression between asphyxiated newborns developing brain injury and healthy newborns**. Thirteen angiogenesis-related proteins showed altered levels. While MMP-9 expression was not different between the two groups, KLK-5, VEGF-C and BDNF still showed reduced expression. Among the proteins enhancing endothelial cell survival, proliferation and migration, the expression of Gal3 and PlGF was still increased; but the expression of NP-1, G6PI, Erb-B3 and FABP-4 was not different, and the expression of heparin-binding epidermal growth factor-like growth factor (HB-EGF) was decreased. Collagen-IV, which is important for vascular stabilization, showed reduced expression. Among the anti-angiogenic proteins, Fib-1C and IGF-BP-6 expression remained decreased. Additionally, IGF-BP-1 and IGF-BP-4 were decreased, reflecting decreased expression of a greater number of anti-angiogenic proteins in these newborns compared to newborns not developing brain injury. Finally, the apoptotic proteins sortilin and FasL remained decreased, while FasR remained increased. When performing a DAVID functional clustering of Gene Ontology Terms using the 49 angiogenesis-related proteins as background and the abovementioned 13 dysregulated proteins as the Gene List, two clusters of proteins stood out, one related to programmed cell death (Fas, FasL, Sortilin) and one related to extracellular signaling activities (Collagen-IV, Fib-1C, KLK-5, IGF-BP-1, IGF-BP-4, IGF-BP-6); four proteins could not be grouped.
(TIF)
