# Supplementary figures and images for: Angiogenesis Dysregulation in Term Asphyxiated Newborns Treated with Hypothermia
Source: PLoS One. 2015 May 21;10(5):e0128028. doi: 10.1371/journal.pone.0128028 (PMC4440713; doi:10.1371/journal.pone.0128028)

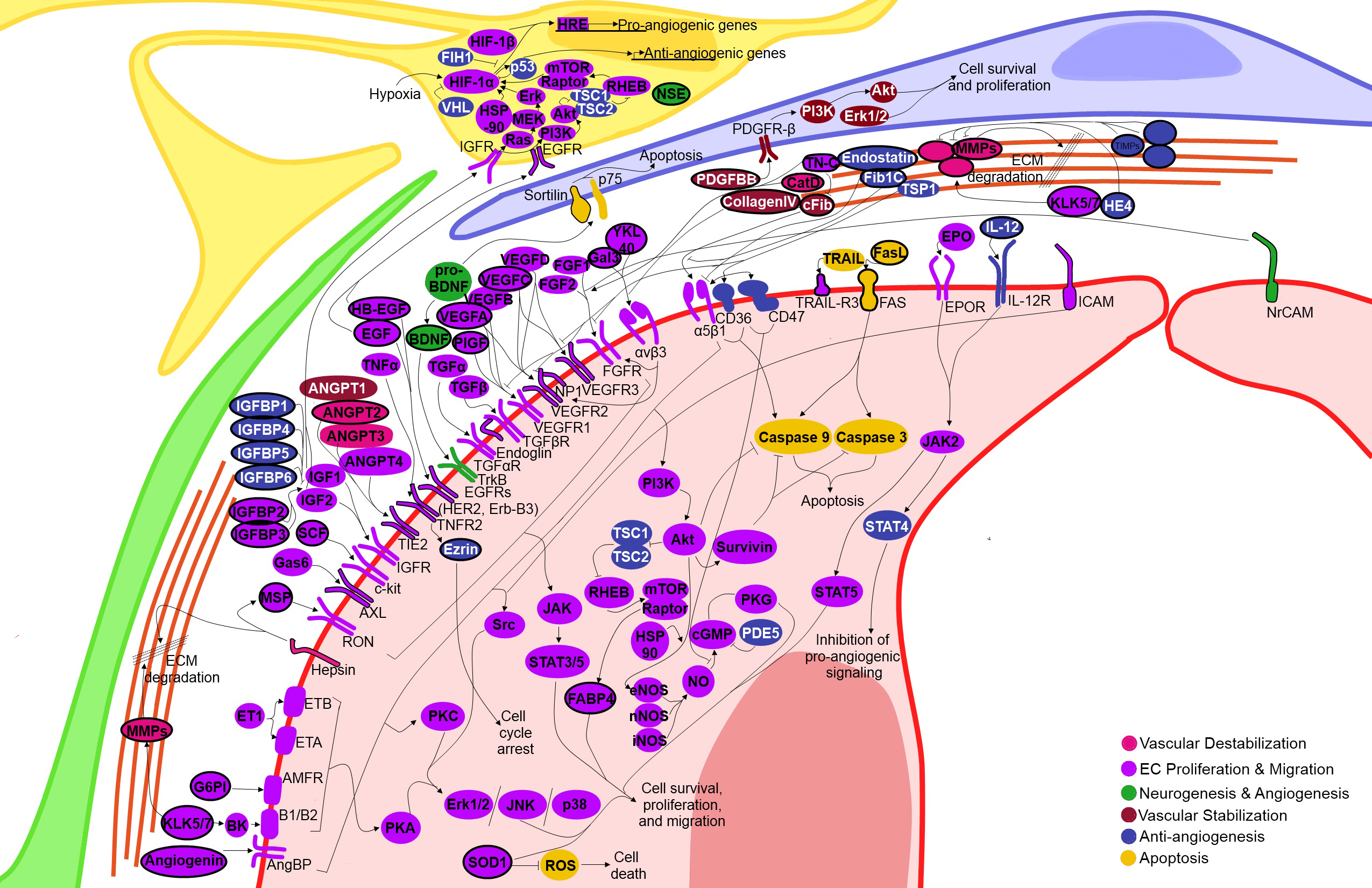

Supplement: S1 Fig — Expression of 49 angiogenesis-related proteins, which were selected based on angiogenic involvement and assay availability, was analyzed. (TIF) [file pone.0128028.s001.tif]
